# Autoimmunity in Syndromes of Orthostatic Intolerance: An Updated Review

**DOI:** 10.3390/jpm14040435

**Published:** 2024-04-20

**Authors:** Clarissa Pena, Abdelmoniem Moustafa, Abdel-Rhman Mohamed, Blair Grubb

**Affiliations:** 1 Department of Internal Medicine, University of Toledo, Toledo, OH 43614, USA; abdelrhman.mohamed@utoledo.edu; 2 Division of Cardiovascular Medicine, University of Toledo, Toledo, OH 43614, USA; abdelmoniem.moustafa@utoledo.edu (A.M.); blair.grubb@utoledo.edu (B.G.)

**Keywords:** adrenergic antibodies, cholinergic antibodies, autonomic dysregulation, POTS, angiotensin II type I antibodies, COVID-19

## Abstract

Orthostatic intolerance is a broad term that represents a spectrum of dysautonomic disorders, including postural orthostatic tachycardia syndrome (POTS) and orthostatic hypotension (OH), as manifestations of severe autonomic failure. While the etiology of orthostatic intolerance has not yet fully been uncovered, it has been associated with multiple underlying pathological processes, including peripheral neuropathy, altered renin–aldosterone levels, hypovolemia, and autoimmune processes. Studies have implicated adrenergic, cholinergic, and angiotensin II type I autoantibodies in the pathogenesis of orthostatic intolerance. Several case series have demonstrated that immunomodulation therapy resulted in favorable outcomes, improving autonomic symptoms in POTS and OH. In this review, we highlight the contemporary literature detailing the association of autoimmunity with POTS and OH.

## 1. Introduction

Orthostatic intolerance is a broad term that represents a spectrum of dysautonomic disorders, including postural orthostatic tachycardia syndrome (POTS) and orthostatic hypotension (OH). The hallmark of orthostatic intolerance is the triggering of symptoms upon standing [1,2].

OH is defined as a decrease in blood pressure of 20 mm Hg for systolic blood pressure or 10 mm Hg for diastolic blood pressure within 3 min of standing [3]. Older age has been associated with the development of OH [4]. POTS is defined as being in an upright posture with a sustained increase in heart rate of 30 beats/minute within 10 min of standing or head-up tilt in the absence of OH. The resulting standing heart rate in POTS patients is generally 120 beats/min [3]. POTS primarily affects women of childbearing age [5,6]. Patients with POTS and OH report debilitating symptoms, including lightheadedness, tachycardia, presyncope, nausea, headache, difficulty concentrating, and memory problems [5].

The relationship between autoimmune disease and orthostatic intolerance is well established. Case reports of patients with autoimmune-related OH have been published. Furthermore, 20% of POTS patients have a diagnosis of a coexisting autoimmune disease, including but not limited to Hashimoto’s thyroiditis, celiac disease, Sjogren’s disease, rheumatoid arthritis, and systemic lupus erythematosus [5,6,7].

Interest in underlying autoimmune process in POTS started decades ago. The initial evidence of autoantibodies (AAbs) in POTS patients was reported by Vernino et al. in 2000 when AAbs targeting ganglionic receptors were identified in 7% of POTS patients while not being found in healthy controls [8]. A study by Wallukat et al. suggested an autoimmune mechanism, supported by isolation of AAbs targeting the beta 2-adrenergic receptor (β2AR), the muscarinic M2 receptor (M2R), and the angiotensin II type 1 receptor (AT1R) [9]. In addition to ganglionic receptor AAbs, identification of beta 1 adrenergic receptor AAbs (β1AR), β2AR AAbs, and muscarinic 3 receptor (M3R) AAbs was reported in 2012 by Yu et al. [10]. More recently, AT1R AAbs were found [11].

The diagnosis of orthostatic intolerance is often preceded by a viral illness or vaccination [12,13,14,15,16]. In the COVID-19 era, orthostatic intolerance was frequently encountered post SARS-CoV-2 infection and vaccination, providing further evidence of an autoimmune etiology [16,17]. In this article, we review the available literature correlating autoimmunity with orthostatic intolerance syndromes. A computerized search in the PubMed, Medline, and Embase databases was performed to retrieve studies with data on orthostatic intolerance and autoantibodies using the search terms orthostatic intolerance, postural orthostatic tachycardia syndrome, orthostatic hypotension, and autoimmunity. Subsequently, a manual search of the reference lists from the retrieved articles was completed to identify additional articles.

## 2. Pathophysiology

### 2.1. Pathophysiology of Orthostatic Hypotension

OH is subdivided into neurogenic and non-neurogenic OH. Neurogenic OH is associated with neurodegenerative disorders, such as multiple system atrophy and Parkinson’s disease; autoimmune diseases; and neuropathy-associated conditions such as diabetes [12,18,19]. Upon standing, there is a decrease in circulating blood volume of approximately 500 mL to 1000 mL. The decreased blood volume leads to a decrease in preload, stroke volume, and blood pressure. When baroreceptors sense decreased stretch due to decreased intravascular volume, compensatory sympathetic activation increases heart rate and vascular tone to mitigate the effect of the decreased circulating blood volume [20,21,22]. In neurogenic OH, there is a lack of increase in vascular tone upon standing due to impairment of norepinephrine release [6].

Non-neurogenic OH is due to different mechanisms, including a decrease in circulating blood volume or medication induced by diuretics and vasodilators [19]. Neurogenic and non-neurogenic OH are differentiated by the difference in heart rate from standing and sitting. In neurogenic OH, the change in heart rate from sitting to standing is less than 15 beats per minute [23,24]. When using heart rate to distinguish between the subtypes of OH, it is important to exclude confounding factors, such as bradyarrhythmias, pacemaker dependence, and atrioventricular nodal blocking agents [25].

### 2.2. Pathophysiology of Postural Orthostatic Tachycardia Syndrome

POTS is theorized to be the culmination of multiple underlying pathological processes, including peripheral neuropathy/denervation, hypovolemia with altered renin–aldosterone levels, and a hyperadrenergic state. The mechanism of peripheral denervation is similar to that of venous pooling with lack of compensatory physiological responses described for OH [12,26].

Regarding hypovolemia, blood volume is reduced in a majority of POTS patients. Reduced stroke volume in the state of hypovolemia is accompanied by compensatory tachycardia to maintain cardiac output [27]. Several studies reported improvement in the severity of POTS symptoms with acute intravascular volume expansion utilizing intravenous saline or desmopressin [28,29,30]. In addition to compensatory tachycardia, hypovolemia activates the renin–angiotensin–aldosterone system (RAAS), enhancing renal sodium and water retention and subsequent volume expansion. Some POTS patients with the hypovolemic subtype have inappropriately high levels of angiotensin II with low levels of renin and aldosterone [31].

In hyperadrenergic POTS, upon standing for 10 min there is an associated increase in systolic blood pressure of 10 mm Hg and plasma norepinephrine levels of 600 pg/mL [32]. Hyperadrenergic POTS also has associated symptoms of palpitations, tachycardia, and anxiety. These POTS patients are particularly sensitive to any agents that increase adrenergic activity at small doses that have not been shown to induce hemodynamic change in the general population [5,33]. Orthostatic tachycardia without hypotension is key for the diagnosis of POTS [3].

#### 2.2.1. Adrenergic Receptors

Adrenergic receptors are G coupled protein receptors. Adrenergic receptors are further divided into alpha 1, alpha 2, beta 1, beta 2, and beta 3 receptors [34,35]. Alpha 1 adrenergic receptors (α1ARs) exert effects on the blood vessels, increase contractility of the left ventricle, and promote coronary artery vasoconstriction [30]. Table 1 summarizes the adrenergic receptor locations and their physiological effects.

The relationship between adrenergic receptor autoantibodies and POTS has been investigated by several studies. The autoantibody-mediated vasodilation mechanism of POTS is apparent when an individual stands. The augmented sympathetic activity leads to orthostatic tachycardia and palpitations. Several studies have demonstrated that AAbs activating β1AR contribute to orthostatic tachycardia, palpitations, and an enhanced adrenergic response [36,37]. Li et al. was able to successfully isolate the human monoclonal antibody that stimulates Beta 2 adrenergic receptors in a patient with orthostatic hypotension and tachyarrhythmias. The effects observed included arteriolar vasodilation, suggesting that the isolated monoclonal antibody has a role in potent vasodilation by altering the normal physiological response to orthostasis [38].

Yu et al. identified autoantibodies against beta adrenergic receptors in five of six patients with OH by use of the enzyme-linked immunosorbent assay (ELISA). These beta adrenergic autoantibodies resulted in activation of protein kinase A, increased contractile activity in cardiac tissue, and altered peripheral vessel contractility demonstrated by a significant dose-dependent vasodilatory effect in animal models [10]. In a clinical study by Li et al., ELISA was used to identify three patients with idiopathic OH and four patients with diabetic OH with more β2AR activation than the healthy controls. The study also demonstrated dose-dependent vasodilation in a rat cremasteric arteriolar assay [39]. In the studies of both Yu et al. and Li et al., the activity of AAbs was blunted by use of propranolol [10,39].

Further studies have demonstrated the presence of AAbs to alpha adrenergic receptors in addition to beta adrenergic receptors. A subsequent study by Li et al. evaluating POTS patients demonstrated the presence of activating β1AR AAbs in all 14 patients and of β2AR autoantibodies in 7 of 14 patients. Evidence of α1AR receptor partial agonist AAbs was also found in POTS patients when their sera infusion caused blunted phenylephrine response in a rat cremaster arteriolar assay [37].

Similar results were found in a study of 17 POTS patients by Fedorowski et al. In this study, 11 of the POTS patients had β1AR AAbs, 12 of the POTS patients had β2AR AAbs, and 8 of the POTS patients had α1AR AAbs [36]. Gunning et al. evaluated 55 POTS patients for adrenergic and muscarinic antibodies. A total of 49 of 55 patients in this study exhibited elevation of α1AR AAbs [40].

Kharrazia et al. measured receptor activity rather than directly measuring AAbs. The receptor activity of all measured receptors—α1AR, β2AR, M2R, and opioid receptor-like 1—was found to be higher in POTS patients compared to controls. Of importance is that the study demonstrated that severity of POTS was correlated strongly with the presence of α1AR [41].

In contrast to most of the studies presented, a study by Hall et al. found no significant difference in 11 antibody levels of adrenergic, muscarinic, angiotensin II, and endothelin between POTS patients and healthy controls [42]. Table 2 summarizes the available evidence for adrenergic antibodies in patients with orthostatic intolerance.

#### 2.2.2. Cholinergic Receptors

Cholinergic receptors are activated by acetylcholine and broadly divided into nicotinic (nAChRs) and muscarinic receptors (mAChRs). The nAChR is found postsynaptically in all autonomic ganglions and at the neuromuscular junction. The mAChR is further categorized into three subtypes, M1, M2, and M3. M1 receptors (M1Rs) are involved in central nervous system transmission. M2 receptors (M2Rs) and M3 receptors (M3Rs) affect exocrine function, gastrointestinal motility, the cardiovascular system, and the airways. Muscarinic receptors are present on the endothelial cells of blood vessels. Although these endothelial muscarinic receptors are not innervated, activation of these receptors by circulating molecules causes vasodilation. In the heart, muscarinic receptors decrease heart rate and slow atrioventricular conduction [2].

In a case study of a patient with OH, the initial presentation was a syncopal event. Subsequent encounters revealed recurrent syncopal events and vital signs consistent with OH. The neurological findings of ptosis and bilateral pupil dilation with diminished pupillary reactivity prompted investigation with a paraneoplastic panel, revealing elevated titers of AChR antibodies. This led to the diagnosis of autoimmune autonomic ganglionopathy (AAG) [6].

In a recent study of 10 POTS patients, 5 patients had elevated M2R AAb levels while none of the controls had elevated M2R AAb activity. The antibody demonstrated a dose-dependent response to increased M2R activation. Furthermore, these antibodies attenuated the response to the M2R agonist oxotremorine. These M2R AAbs may contribute to excessive orthostatic tachycardia due to enhanced withdrawal of vagal tone upon standing [43]. Another study demonstrated a significant association between gastrointestinal symptoms in patients with POTS and levels of mAChR autoantibodies. This is particularly important as it highlights the correlation between the presence of AAbs and the clinical manifestations of POTS [44]. Several studies reported the presence of nicotinic antibodies in patients with orthostatic intolerance. A correlation between the seropositive patients and other dysautonomic manifestations, such as neurogenic bladder and the sicca complex, was demonstrated [8,45,46,47].

Watarai et al. provided further evidence of the autoimmune basis of POTS by examining the presence of AAbs in POTS patients and patients with neurally mediated syncope. mAChR AAbs were found in 10 of the POTS patients. They occurred with greater frequency in the POTS patients compared to the neurally medicated syncope patients [48].

While autoantibody presence can hint at the autoimmune etiology of orthostatic intolerance, the presence of AAbs is not always clinically significant. Bryarly et al. demonstrated that very low levels and low levels of gACh could be found in the sera of POTS patients and controls. Furthermore, there was no clinical difference between the seropositive POTS patients and the seronegative POTS patients [49].

Table 3 summarizes the studies that investigated anticholinergic antibodies in patients with orthostatic intolerance.

#### 2.2.3. Angiotensin II Type I Receptors

Inappropriately high levels of angiotensin II with low levels of renin and aldosterone in POTS patients have been previously reported. Despite the high levels of angiotensin II, the pressor response is reportedly absent as patients have normal blood pressure. It has been postulated that the blunted pressor response is due to the persistently high levels of angiotensin II as well as reduced activity of angiotensin converting enzyme 2 (ACE2) [31,52,53].

More recently, the role of antibodies against A1TR has emerged. AT1R is another G-coupled protein similar to adrenergic receptors. In a pilot study by Yu et al., serum samples from 17 patients with POTS, 6 patients with recurrent vasovagal syncope (VVS), and 10 controls were obtained. This study demonstrated significant AT1R activity using separated IgG from POTS serum samples as compared to VVS and healthy controls. This AT1R activity was reduced after using losartan, an AT1R blocker. The results of this study provided evidence for the presence of AT1R antibodies in POTS patients [11].

#### 2.2.4. COVID-19 and POTS

The COVID-19 pandemic has generally been associated with respiratory symptoms in the acute phase. However, there have been a variety of symptoms associated with post-acute COVID-19 infection. Long COVID is a term that includes ongoing symptomatic COVID-19 (4 to 12 weeks) and post-COVID-19 syndrome (>12 weeks) that are not explained by an alternative diagnosis [54]. Case reports have described a new onset of autonomic dysfunction symptoms with features of POTS/inappropriate sinus tachycardia in the post-acute phase of COVID-19 infection [55,56]. The underlying cause of dysautonomia post-COVID-19 infection is not well understood. However, a viral infection by SARS-CoV-2 triggering autoimmune response and direct neurotoxic effects has been suggested as an underlying cause for developing post-COVID-19 POTS [57,58]. Wallukat et al. conducted a study in which 31 patients with POTS and COVID-19 were examined. All patients had positive autoantibodies ranging from two to seven. The autoantibodies that were most frequently positive were β2AR, M2R, and AT1R. The presence of β2AR exerted a positive chronotropic effect, the presence of M2AR exerted a negative chronotropic effect, and AT1R exerted a positive chronotropic effect on their targets [9].

A retrospective case series by Blishteyn et al. evaluated patients with no history of chronic orthostatic intolerance. They found evidence of orthostatic intolerance following SARS-CoV-2 infection. In the study there were 15 POTS patients, 2 patients with OH, and 3 patients with neurocardiogenic syncope. Four of the twenty patients had elevated autoimmune/inflammatory markers. Seventeen of the patients had residual autonomic effects that negatively impacted their lives 6 months following SARS-CoV-2 infection. For 12 of the patients, orthostatic intolerance was severe enough to preclude a return to work [16].

Not only does contracting SARS-CoV-2 infection confer the possibility of developing long COVID and subsequent development of POTS, but recipients of the SARS-CoV-2 vaccine have also been shown to develop POTS at a higher rate. The proposed explanation is that an immunological response was elicited by the administration of the vaccination, resulting in similar symptoms to long COVID. The study compared two cohorts, one whose members received the SARS-CoV-2 vaccine and another whose members were positive for SARS-CoV-2 infection to evaluate the diagnosis of POTS both before and after exposure to the vaccine or infection. It was determined that the SARS-CoV-2 vaccine was associated with a statistically significant increase in the development of POTS; however, this increase was less than the development of POTS following SARS-CoV-2 infection [14].

#### 2.2.5. Antiphospholipid and Antinuclear Autoantibodies

Blitshteyn performed a retrospective review of POTS patients to evaluate comorbid autoimmune disorders and the presence of AAbs. Autoimmune disorders were present in 20/100 patients, including Hashimoto’s thyroiditis (11/100), antiphospholipid syndrome (5/100), rheumatoid arthritis (4/100), celiac disease (3/100), systemic lupus erythematosus (2/100), and Sjögren’s syndrome (2/100). Antinuclear autoantibodies (ANAs) were positive in 25/100 POTS patients, while antiphospholipid autoantibodies (aPLs) were positive in 7/100 POTS patients. A higher prevalence of ANA AAbs and aPL AAbs were found in patients with POTS compared to the general population. The presence of Hashimoto’s thyroiditis, systemic lupus erythematosus, and rheumatoid arthritis was found to be statistically significantly higher in POTS patients compared to the general population [59].

A retrospective case series by Schofield et al. identified 15 patients with antiphospholipid syndrome and orthostatic intolerance. Regarding orthostatic intolerance, 8/15 had POTS, 8/15 had neurocardiogenic syncope, and 3/15 had orthostatic hypotension. Comorbid autoimmune conditions included rheumatoid arthritis (1/15), systemic lupus erythematosus (2/15), and celiac disease (1/15). Two of the POTS patients failed to improve with standard treatment for antiphospholipid syndrome but subsequently responded well to IVIG [60].

#### 2.2.6. Treatment

In addition to orthostatic intolerance being associated with increased mortality, there is a significant impact on quality of life that can prove devastating [12,61]. The initial approach to managing POTS/OH is usually non-pharmacological, including avoidance of triggers (exposure to heat and prolonged standing), graded exercise training, using waist-high compression stockings, and liberal salt and fluid intake [27]. If the non-pharmacological approach is proven to be inadequate, several off-label medications that have demonstrated symptomatic improvement will be administered [12]. These medications include fludrocortisone, ivabradine, beta blockers, midodrine, and pyridostigmine. These medications can be utilized as monotherapy or more often as a combination therapy [12]. For patients with OH, there has been success in treatment with l-threo-3,4-dihidroxyphenylserine (l-DOPS), a synthetic catecholamine that converts to norepinephrine when ingested orally [7,62,63]. Nonetheless, one-third of POTS patients remain symptomatic despite escalation of medical therapy [64]

The question arose whether the available knowledge pertaining to the autoimmunity in orthostatic intolerance patients would predict a role for immune modulation therapeutics in patients with refractory orthostatic intolerance and evidence of existing AAbs. Several case reports and case series demonstrate that immune modulation agents have a possible role in the treatment of orthostatic intolerance in patients who have symptoms refractory to the commonly used pharmacological and non-pharmacological treatments.

Pitarokoili et al. reported a case study in which a female patient with Marfan’s syndrome developed POTS 2 weeks following administration of pneumococcal vaccination. Antibodies against adrenergic β1 and β2, muscarinic M2 and M4, and nociceptin-like receptors were positive. She was treated with intravenous immunoglobulin (IVIG), which resulted in improvement of orthostatic symptoms and decreased AAb activity of adrenergic β1 and β2, muscarinic M2 and M4, and nociceptin-like receptors. Maintenance therapy was changed to subcutaneous immunoglobulin. This patient was also able to decrease the dose of clonidine and discontinue midodrine [65]. Another case report for a woman with POTS and mast cell activation syndrome showed improvement in her tachycardia and sudomotor function after 10 IVIG treatments [66]. 

A large retrospective study by Schofield et al. evaluated the use of IVIG in patients with refractory autoimmune dysautonomias. After being treated with IVIG for at least 3 months, patients experienced improvement in dysautonomic symptoms. The study also demonstrated that the presence of aPL AAbs and Sjögren AAbs correspond to a positive response to IVIG [67] 

A case series investigated the role of IVIG treatment in POTS patients. Autoimmune testing revealed that all six patients had AAbs against α1AR, while four of six patients had AAbs against mAChR. After 6 months of IVIG treatment, all patients reported less fatigue, improvement of orthostatic intolerance, and improvement of sudomotor function. Five of six patients described improved exercise tolerance and gastrointestinal symptoms [51]. 

Kesterson et al. presented a case series of seven patients with POTS treated with subcutaneous immunoglobulin or plasmapheresis. Two patients had positive nAChR at low titers; one patient had elevated adrenergic, muscarinic, AT1R, and endothelin I receptor antibodies; and two patients did not have any identifiable AAbs. The outcomes showed significant improvement in orthostatic symptoms and functional abilities measured by questionnaires preimmunotherapy, 3 months post-treatment, and 12 months post-treatment. Reduction or discontinuation of oral POTS medications was reported among the patients [68].

While IVIG has shown positive response with improvement of symptoms in POTS patients, there are limited data of any benefit on OH patients in the setting of autoimmune autonomic ganglionopathy. A case series explored three patients who did not respond to the conventional methods of fludrocortisone, midodrine, vasopressin, and erythropoetin; plasmapheresis alone; and IVIG alone. They were treated with combination prednisone and mycophenolate mofetil for 6 months followed by five cycles of plasmapheresis. After the course of treatment, OH resolved and mean antibody levels decreased. These results indicate that patients with refractory autoimmune autonomic ganglionopathy may benefit from a multimodal approach to therapy to treat OH [50].

To date, there has been only one randomized control trial, iSTAND, that has evaluated the efficacy and safety of IVIG in POTS patients with moderate to severe symptoms and evidence of autoimmunity either by the presence of AAbs or the coexistence of defined autoimmune diseases. Thirty participants were randomized to receive either IVIG or albumin infusions. COMPASS-31 scores were used to assess symptom response to IVIG and albumin infusions. The iSTAND trial, while not showing a significant difference in symptom outcomes between the IVIG and albumin groups, highlights the challenges in determining optimal treatment strategies for POTS patients with evidence of autoimmunity. The fact that the authors suggested that volume expansion could have been treated with IVIG and albumin emphasizes the complexity of managing these patients and the need for individualized approaches [69]

## 3. Discussion

This review highlights the contribution of AAbs in symptoms triggered by upright position. AAbs targeting adrenergic receptors cause dose-dependent vasodilation by activating β2AR and partial α1AR antagonism. Impaired vasoconstriction will be paired with tachycardia and palpitations upon standing [10,37,40]. Moreover, β1AR AAbs enhance sympathetic response, causing excessive tachycardia and palpitations with upright position [36,37]. M2R AAbs suppress the function of M2R, with subsequent increased vagal tone withdrawal upon standing [43]. Furthermore, recently discovered AAbs against AT1R may play a role in the state of hypovolemia and RAAS imbalance in a subgroup of POTS patients [11]. In a study by Gunning et al., the detection of α1AR-AAbs in POTS patients was coupled with significant elevation of several cytokines compared to control subjects, shedding light on autoimmunity in POTS and the autoinflammatory state in this disease [70].

### 3.1. Importance of a Reliable Methodology

In order to be able to effectively target the patients with an autoimmune component of POTS, AAbs need to be properly identified. The studies presented in this review use various methods for the identification of AAbs. Hall et al. provided evidence that there was no significant difference between controls and POTS patients by using ELISA [42]. This finding is contrary to what other studies investigating orthostatic intolerance have determined. It was postulated by the authors that this discrepancy was due to theirs being the first study to evaluate POTS with a control group. Several of the studies completed prior to Hall et al. did have a control group for orthostatic hypotension and demonstrated significant differences in AAbs between patients with OH and the controls [10,39]. However, it should be noted that the size of the studies was smaller, which may affect the reliability of the studies. Future studies should include a larger quantity of participants to determine if ELISA can reliably be used as a methodology to identify AAbs. Furthermore, it may be of interest to identify AAbs with ELISA and a different assay to compare detection of AAbs with different assays in syndromes of orthostatic intolerance.

### 3.2. Individualizing Treatment Plans

The variety of treatment options for orthostatic intolerance serve as a reminder that treatment is not straightforward; an individualized approach is needed. This is particularly evident with the use of IVIG, as the iSTAND trial demonstrated no significant difference in symptoms when compared to the control group [51] In order to formulate an individualized approach, the exact role of AAbs in orthostatic intolerance needs to be further elucidated. Much of the available literature reports the effects of AAb stimulation on receptors; however, little is known about the mechanism by which AAbs exert their effects.

While there is a dearth of studies explaining the mechanism of AAbs in orthostatic intolerance, there are a few studies that provide valuable insights. Deng et al. investigated M2R-AAbs in a rabbit model. Treatment of M2R-immunized rabbits with low-level tragus stimulation (LLTS) was performed to stimulate the vagus nerve. LLTS treatment resulted in blunting of postural tachycardia, increased acetylcholine secretion, and improved the attenuated chronotropic heart rate response. This study provides insight into the mechanism of M2R AAbs, showing that by increasing the production of acetylcholine, the effects of M2R AAbs can be overcome. Further insight into the mechanism is provided with the noted decrease in inflammatory cytokines following treatment with LLTS [71] Guo et al. performed a similar study with alpha adrenergic receptors and beta adrenergic receptors in a rabbit model. The results indicated that there was increased release of acetylcholine and elevated inflammatory markers [72].

Stavrakis et al. provided evidence of the mechanism of AAb-mediated POTS by evaluating transcutaneous vagal stimulation in a randomized control trial. The results were decreases in β1AR and α1AR autoantibodies, improvement in cardiac autonomic function, and a decrease in serum inflammatory cytokines. From a POTS symptom standpoint, patients experienced less sudomotor stimulation and decreased orthostatic tachycardia [73]. Of particular importance is the noted decrease in adrenergic AAb production. These studies illuminate the mechanism by which AAbs exert their effects and demonstrate a cost-effective method for treatment of autoimmune-mediated POTS that can be incorporated into an individualized treatment plan. Future studies are needed to understand how increased parasympathetic stimulation leads to decreased AAb production.

Once identified, it is important to clinically correlate symptoms with AAbs. Bryarly et al. demonstrated that the mere presence of very low and low levels of an antibody does not indicate clinical significance [49]. This is important, as further costs to the patient can be avoided if the clinician recognizes that there is no need for further immune workup with low titers. This has implications for treatment, as in such a case the patient would not be classified according to the autoimmune etiology of orthostatic intolerance and other treatment options could be explored.

A universal definition of what constitutes the autoimmune etiology of POTS by titer levels would be useful. The application of a universally agreed upon definition would directly impact studies. The iSTAND trial evaluated patients with suspected autoimmune etiology of POTS; however, the study failed to mention what the titer levels were for patients. The patients who received IVIG in the study could have had low titer levels, resulting in no difference to the control group [69].

As previously mentioned, there are several postulated mechanisms for POTS. POTS patients have reported a delay in diagnosis, likely due to the complexities associated with understanding the diagnosis of POTS [5]. While the focus of this review is on studies related to autoimmune-mediated orthostatic intolerance, the presence of AAbs is not necessary for the diagnosis of orthostatic intolerance. If general practitioners are cognizant of the association of orthostatic intolerance with autoimmune etiology, this may prompt them to consider the diagnosis of POTS or obtain an early referral to a specialist, such as a neurologist or a cardiologist, who can confirm the diagnosis and provide further management. With evidence that many patients suffering from long COVID can develop autoimmune-mediated orthostatic intolerance, prompt recognition is important as it can expedite appropriate treatment [9]. By recognizing the autoimmune component of both OH and POTS, an investigation can be quickly started to identify any possible associated autoimmune disorder and expedite early treatment with immunomodulating therapies.

## 4. Conclusions

The review provides a comprehensive overview of the existing evidence linking orthostatic intolerance to autoimmunity. Many studies have shown increased levels of AAbs in patients with orthostatic intolerance when compared to controls. The only randomized control study evaluating IVIG for autoimmunity failed to show the benefit of IVIG over standard methods of volume expansion. This highlights the complexity of managing orthostatic intolerance and emphasizes the need for larger randomized control trials to explore the specificity of AAbs. While the effects of AAbs on receptors are known, further studies evaluating the mechanism could aid in the development of more treatment options.

## Figures and Tables

**Table 1:** Adrenergic receptors and physiological effects.

Receptor	G Protein	Location	Physiological Effects
α1	Gq	Smooth muscles of blood vessels, heart, urinary tract	Arterial and venous constriction, increased ventricular contractility, urinary retention
α2	Gi	Central nervous system, presynaptic sympathetic nerves	Constriction of smooth muscle
β1	Gs	Heart	Increased heart rate, increased ventricular contractility
β 2	Gs	Lung, genitourinary smooth muscle, gastrointestinal tract, platelets	Increased ventricular contractility, increased heart rate Blood vessel dilation Decreased platelet aggregation
β3	Gi Gs	Heart, genitourinary tract, adipose tissue	Decreased cardiac contractility, increase lipolysis

α1AR: alpha 1 adrenergic receptor, α2AR: alpha 2 adrenergic receptor, β1AR: beta 1 adrenergic receptor, β2AR: beta 2 adrenergic receptor, β3AR: beta 3 adrenergic receptor.

**Table 2:** Adrenergic antibodies in patients with orthostatic intolerance.

Author	Year	Group of Patients Tested	Receptor-Associated Autoantibodies	Number of Patients with Positive Autoantibodies/Number of Total Patients, Number of Controls with Positive Antibodies/Number of Total Controls	Comments
Yu et al. [10]	2012	Idiopathic OH	β1/2AR	5/6, 0/10	
Li et al. [39]	2012	10 idiopathic OH and 10 diabetic patients with OH	β2AR	7/20, 0/10	
Li et al. [37]	2014	POTS POTS POTS	β1AR β2AR α1AR	14/14, 0/10 7/14, 0/10 14/14, 0/10	
Fedorowski et al. [6]	2016	POTS POTS POTS	α1AR β1AR β2AR	8/17, 0/11 11/17, 0/11 12/17, 0/11	
Gunning et al. [40]	2019	POTS POTS	α1AR	49/55, N/A	
Hall et al. [42]	2022	POTS Control	ELISA: AT1R ETR α1AR α2AR β1AR β2AR M1R M2R M3R M4R M5R	41/116, 22/81 24/116, 15/81 114/116, 81/81 31/116, 22/81 11/116, 7/81 9/116, 5/81 N/A N/A 24/116, 23/81 28/116, 15/81 N/A	No statistically significant difference in 11 autoantibody levels (adrenergic, muscarinic, angiotensin II, and endothelin) was found between POTS patients and healthy controls

α1AR: alpha 1 adrenergic receptor, AT1R: angiotensin receptor, β1AR: beta 1adrenergic receptor, β2AR: beta 2 adrenergic receptors ETR: endothelin receptor, M1R: muscarinic 1 receptors, M2R: muscarinic 2 receptors, M3R:muscarinic 3 receptors; M4R: muscarinic 4 receptors; M5R muscarinic 5 receptors OH: orthostatic hypotension, POTS: postural orthostatic tachycardia syndrome.

**Table 3:** Muscarinic receptor antibodies in patients with orthostatic intolerance.

Author	Year	Receptor	Patient Population	Number of Patients with Positive Antibodies/Number of Total Patients, Number of Controls with Positive Antibodies/Number of Total Controls	Comments
Vernino et al. [8] Sandroni et al. [39]	2000 2004	A3-AChR Ab Ganglionic AChR	POTS Orthostatic intolerance, autonomic neuropathy	6/67, N/A Compared 19 seropositive with 87 seronegative patients	Seropositive patients are more likely to have orthostatic hypotension with other cholinergic symptoms like the sicca complex or GI symptoms
Gibbons et al. [50]	2008	AChR	Autoimmune autonomic ganglionopathy	3/3, N/A	Three patients with dysautonomia and nicotine receptor antibody refractory to medical treatment who responded to immunomodulatory therapy
McKeon et al. [45]	2009	A3-AChR Ab	Paraneoplastic neurological ganglionopathy	155/15,000 (1%) with positive titers were examined; 13 had pan-dysautonomia, 5 had orthostatic hypotension only	High antibody values of 1.00 nmol/L were associated with pan-dysautonomia
Gibbons et al. [47]	2009	AChR	Autoimmune autonomic ganglionopathy	8/8, N/A	Higher antibody titers were associated with more severe orthostatic hypotension
Yu et al. [10]	2012	M2/M3 receptor Ab	Idiopathic OH	6/6, 0/10	Serum from patients caused dose-dependent vasodilation in rat cremaster arteriole
Li et al. [39]	2012	M3R	10 idiopathic OH and 10 diabetic patients with OH	13/20, 0/10	Serum from patients caused vasodilation in rat cremaster arteriole. The effect was dose-dependent and inhibited by adding atropine
Li et al. [43]	2022	M2R	POTS	5/10, 0/10	These antibodies suppressed the function of M2R in a dose-dependent fashion and may contribute to excessive orthostatic tachycardia due to enhanced withdrawal of vagal tone upon standing
Sunami et al. [44]	2022	mAChR	POTS	N/A	Significant association between gastrointestinal symptoms in patients with POTS disease and level of mAChR autoantibodies
Vernino et al. [8] McKeon et al. [45] Sandroni et al. [46] Gibbons et al. [47]	2000 2009 2004 2009	nAChR	OI		Found a correlation between the seropositive patients and other dysautonomic manifestations, such as neurogenic bladder and the sicca complex
Fedorowski et al. [6]	2022	AChR	OH	N/A, N/A	Case study where OH was recognized as a part of autoimmune autonomic neuropathy
Watari et al. [48]	2018	AChRa3 AChRb4	POTS	8/34, 1/34 2/34, 0/34	
Rodriguez et al. [51]	2021	mAChR	POTS	4/6, N/A	Improvement of symptoms after IVIG treatment

AChR: cholinergic receptor, GI: gastrointestinal, mAChr: muscarinic receptor, M2R:muscarinic 2 receptor, M3R: muscarinic 3 receptor, nAChR: nicotinic receptor, OH: orthostatic hypotension, OI: orthostatic intolerance, POTS: postural orthostatic tachycardia syndrome.

## Data Availability

No new data were created or analyzed in this study. Data sharing is not applicable to this article.
